# Root GS and NADH-GDH Play Important Roles in Enhancing the Ammonium Tolerance in Three Bedding Plants

**DOI:** 10.3390/ijms23031061

**Published:** 2022-01-19

**Authors:** Jinnan Song, Jingli Yang, Byoung Ryong Jeong

**Affiliations:** 1 Department of Horticulture, Division of Applied Life Science (BK21 Four Program), Graduate School of Gyeongsang National University, Jinju 52828, Korea; jinnansong93@gmail.com (J.S.); yangmiaomiaode@gmail.com (J.Y.); 2 Institute of Agriculture and Life Science, Gyeongsang National University, Jinju 52828, Korea; 3 Research Institute of Life Science, Gyeongsang National University, Jinju 52828, Korea

**Keywords:** nitrogen use efficiency (NUE), ammonium toxicity, salvia, petunia, ageratum, photosynthesis, nitrogen-carbohydrate distributions

## Abstract

Ammonium is a paradoxical nutrient because it is more metabolically efficient than nitrate, but also causes plant stresses in excess, i.e., ammonium toxicity. Current knowledge indicates that ammonium tolerance is species-specific and related to the ammonium assimilation enzyme activities. However, the mechanisms underlying the ammonium tolerance in bedding plants remain to be elucidated. The study described herein explores the primary traits contributing to the ammonium tolerance in three bedding plants. Three NH_4_^+^:NO_3_^−^ ratios (0:100, 50:50, 100:0) were supplied to salvia, petunia, and ageratum. We determined that they possessed distinct ammonium tolerances: salvia and petunia were, respectively, extremely sensitive and moderately sensitive to high NH_4_^+^ concentrations, whereas ageratum was tolerant to NH_4_^+^, as characterized by the responses of the shoot and root growth, photosynthetic capacity, and nitrogen (amino acid and soluble protein)-carbohydrate (starch) distributions. An analysis of the major nitrogen assimilation enzymes showed that the root GS (glutamine synthetase) and NADH-GDH (glutamate dehydrogenase) activities in ageratum exhibited a dose-response relationship (reinforced by 25.24% and 6.64%, respectively) as the NH_4_^+^ level was raised from 50% to 100%; but both enzyme activities were significantly diminished in salvia. Besides, negligible changes of GS activities monitored in leaves revealed that only the root GS and NADH-GDH underpin the ammonium tolerances of the three bedding plants.

## 1. Introduction

Nitrogen (N) is a vital factor influencing plant growth and agricultural productivity; sufficient nitrogen fertilization is necessary for a reliable yield and quality of plants [1,2]. However, an excessive supplementation of nitrogen fertilizers caused not only severe nitrogen pollutions worldwide, but also decreased the nitrogen-use efficiency (NUE) by plants [3]. For instance, foliage turning to dark green abnormally with physiological dysfunction, and lateral root growth and elongation inhibited causing lodging, are usually observed with nitrogen overdose.

Ammonium (NH_4_^+^) and nitrate (NO_3_^−^) are two principal inorganic N forms for plant absorption and assimilation. Both can be taken up and utilized through the root parts, but the energetic and biochemical processes for the acquisition of the two inorganic N forms have been characterized to be dramatically different [4]. Intensive nitrate fertilization results in NO_3_^−^ accumulation, which presents environmental and human health hazards [5]. NO_3_^−^ assimilation by plants requires more energy than NH_4_^+^ assimilation, which conferred NH_4_^+^ as the predominant N source for plants.

Paradoxically, only a few plant species grow better when NH_4_^+^ is applied as the exclusive N source. Furthermore, plant growth and development were suppressed when subjected to millimolar concentrations of NH_4_^+^, which are considered as a key factor in determining the plant species richness [6]. This phenomenon has been well identified as ammonium toxicity, accompanied by several physiological and biochemical changes in plants. Many toxicity symptoms developed by plants suffering from high NH_4_^+^ concentrations were reported; for instance, decreased plant growth and declined yield, together with certain visual detrimental signs, such as leaf chlorosis and stunted root growth [7,8]. As a consequence, the photosynthetic capacity and carbohydrate stock were seriously disrupted during this toxicity process.

These ammonium toxicity symptoms have evoked numerous hypotheses on the cause: depletion of carbon supply [8,9], energy overconsumption for futile ammonium cycling [8,10], and a recently elucidated mechanism of acidic stresses produced by disordered pH regulation [11]. Nonetheless, the information linked with the N assimilation pathways to explain the ammonium tolerances, especially in bedding plants, remains scarce. Although ammonium toxicity appears to be universal, the threshold of toxicity symptom developments differs widely according to the plant species, including bedding plants, which have a pronounced variation in the ammonium tolerance.

Fortunately, it has been well demonstrated that NH_4_^+^ in higher plants is assimilated into amino acids via two conserved routes. NH_4_^+^ is catalyzed through glutamine synthetase (GS) to form glutamine, which is required in a reaction by glutamate synthetase (GOGAT) to synthesize glutamate; this route was named the GS/GOGAT cycle [12,13]. Additionally, NH_4_^+^ can also be transformed into glutamine by glutamate dehydrogenase (GDH), which plays an alternative role to enhancing the ammonium tolerance [14,15]. Furthermore, the priorities of NH_4_^+^ assimilation pathways differ among species due to the specificity and complexity of plant habitation: GDH activity is more important than GS activity for ammonium detoxification in submerged macrophytes [16]; while a higher GS activity in certain vegetables and crops, such as lettuce [15] and sorghum [17], increased the NH_4_^+^ tolerance.

Previous studies attempted to figure out a fertilization strategy by supplying an optimal ratio of NH_4_^+^:NO_3_^−^ for their given bedding plants, neglecting the responses of major enzymes involved in the N assimilation pathways to an increasing NH_4_^+^ supply [18,19,20]. Consequently, no consensus was built on which traits conferred the ammonium tolerance in bedding plants. On the other hand, a substantial amount of NH_4_^+^ was locally incorporated when it was taken up in the roots, then the remainder was transported to the aboveground parts [21]. However, most of the studies devoted to the NH_4_^+^ tolerance mechanisms failed to separate the roots and shoots or to clearly point out the tested plant tissues.

Thus, the study undertaken herein was designed and concentrated to investigate the key traits in determining the NH_4_^+^ tolerance in bedding plants. To this end, we examined the differences of the NH_4_^+^ tolerance in three bedding plants, and identified the NH_4_^+^-tolerant and -sensitive species, as evidenced by the responses of growth attributes, photosynthetic capacity, carbohydrate (starch), and nitrogen (free amino acid and soluble protein) stock to the increasing NH_4_^+^ concentrations. In addition, we monitored and analyzed the changes of major enzyme activities in the NH_4_^+^ metabolism pathways (GS, GOGAT, GDH) both in leaves and roots in response to increasing NH_4_^+^ concentrations, for a better understanding on the potential relationship between NH_4_^+^ tolerance and NH_4_^+^ assimilation in bedding plants.

## 2. Results

### 2.1. NH_4_^+^ Tolerances in Three Bedding Plants

#### 2.1.1. Shoot Growth Attributes as Affected by the NH_4_^+^:NO_3_^−^ Ratio

**Figure 1:**
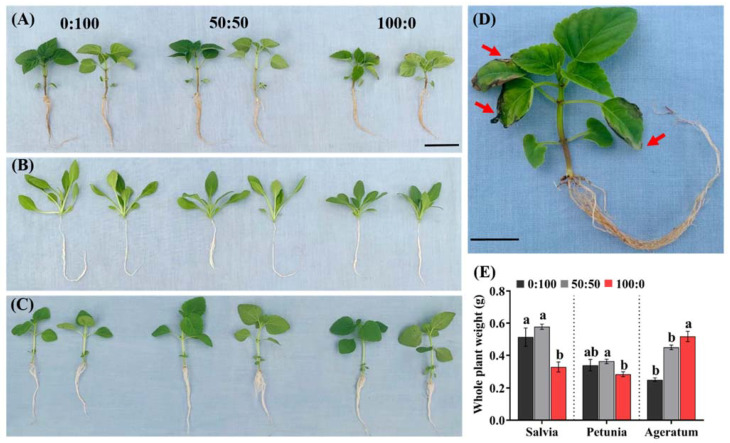
Effects of the three NH_4_^+^:NO_3_^−^ ratios on the growth of (**A**) salvia, (**B**) petunia, and (**C**) ageratum; each plant pair with similar growth represent exposure to the same NH_4_^+^:NO_3_^−^ ratio; (**D**) Enlarged image of a representative salvia plant treated with 100:0 NH_4_^+^:NO_3_^−^ show ammonium toxicity symptoms; (**E**) Whole plant weights of salvia, petunia, and ageratum exposed to the three NH_4_^+^:NO_3_^−^ ratios. Data are the means of six independent replicates ±SD; significant differences at *p* less than 0.05 are denoted by different letters according to the one-way ANOVA (Duncan’s multiple range test). Scale bars in (**A**,**D**) represent 3 cm and 1 cm, respectively. Red arrows point to the typical ammonium toxicity symptoms.

The shoot growth attributes were remarkably affected by the different NH_4_^+^:NO_3_^−^ ratios, regardless of the species. Furthermore, the three bedding plants possessed distinct physiological behaviors in response to the increasing NH_4_^+^ supply. In a whole, with the exception of ageratum, a mixed application of NH_4_^+^ and NO_3_^−^ conferred better growth and yield in comparison with sole NH_4_^+^ or NO_3_^−^ nutrient supply. More importantly, high NH_4_^+^ concentrations most significantly restricted the growth and development of salvia (Figure 1A), followed by those of petunia, as presented in Figure 1B; ageratum displayed the opposite responses where a high NO_3_^−^ concentration suppressed, while sole NH_4_^+^ nutrient supply promoted, the growth (Figure 1C).

In addition, as expected, it was noteworthy that salvia supplied solely with NH_4_^+^ developed ammonium toxicity symptoms, as characterized by the unhealthy leaves with chlorosis, necrosis, and burned tips accompanied by stunted roots (Figure 1D).

Concomitantly, the influences of the different NH_4_^+^:NO_3_^−^ ratios on certain other growth parameters of the three bedding plants were also analyzed, including the shoot length, leaf length, width and area, and total root length (Figure 1E; Table 1). Outstandingly, for salvia, the whole plant weight of solely NH_4_^+^-fed plants declined 35.3% and 43.1%, respectively, compared to the plants fed solely with NO_3_^−^ and with 50:50 NH_4_^+^:NO_3_^−^; 5.88% and 22.2% declines in the whole plant weight were observed for petunia, respectively, when the same comparisons were conducted (Figure 1E ‘Salvia part’ and ‘Petunia part’); whereas the whole ageratum weight increased by 80% and 108% respectively when the NH_4_^+^:NO_3_^−^ ratio increased from 0% to 50% and 100% (Figure 1E ‘Ageratum part’). The *F*-test results revealed that the NH_4_^+^:NO_3_^−^ ratio considerably affected the shoot length, leaf width and area, as well as the total root length; there was a strong interaction between the species and NH_4_^+^:NO_3_^−^ ratios on all the growth attributes mentioned (Table 1).

#### 2.1.2. Root Morphology Parameters as Affected by the NH_4_^+^:NO_3_^−^ Ratios

**Figure 2:**
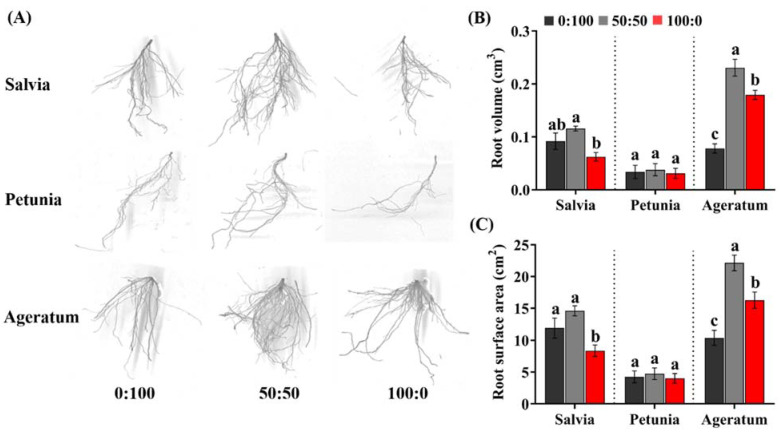
Effects of the three NH_4_^+^:NO_3_^−^ ratios on the growth and development of fine roots of salvia, petunia, and ageratum; (**A**) scanned images of fine roots of salvia, petunia, and ageratum cultured with different NH_4_^+^:NO_3_^−^ ratios; (**B**) root volume (cm^3^) and (**C**) root surface area (cm^2^) of salvia, petunia, and ageratum as affected by the three NH_4_^+^:NO_3_^−^ ratios; data are the means of six independent replicates ±SD; significant differences at *p* less than 0.05 are denoted by different letters according to the one-way ANOVA (Duncan’s multiple range test).

The root morphological traits are associated with the soil and plant nitrogen (N) availability, so we investigated the responses of roots to the different NH_4_^+^:NO_3_^−^ ratios immediately after harvest. All root morphological parameters analyzed herein significantly varied according to the species and NH_4_^+^:NO_3_^−^ ratio. Apparently, the plants cultured with 50:50 NH_4_^+^:NO_3_^−^ showed a larger and deeper root system, as observed by the enhanced branching and density. Salvia and petunia plants cultured with 100:0 NH_4_^+^:NO_3_^−^ possessed smaller roots relative to those cultured with 0:100 NH_4_^+^:NO_3_^−^, whereas larger roots were formed by ageratum plants grown with 100:0 NH_4_^+^:NO_3_^−^ (Figure 2A).

Consistent with the scanned images of the roots, distinguished tendencies of root volume and root surface area among different species and in response to the different NH_4_^+^:NO_3_^−^ ratios were also observed. As presented in Figure 2B,C, salvia plants treated with 100:0 NH_4_^+^:NO_3_^−^ always displayed the lowest root volume and root surface area, which diminished 31.94% and 29.66%, respectively, compared to those of salvia plants grown with 0:100 NH_4_^+^:NO_3_^−^; contrastingly, ageratum plants treated with 100:0 NH_4_^+^:NO_3_^−^ sharply gained respectively 1.29-fold and 56.87% elevation of the root volume and root surface area than compared to those cultured with 0:100 NH_4_^+^:NO_3_^−^. Additionally, neither the root volume nor the root surface area displayed remarkable variations in petunia in response to the different NH_4_^+^:NO_3_^−^ ratios.

### 2.2. Further Confirmations of the NH_4_^+^ Tolerance in the Three Bedding Plants

#### 2.2.1. Effects of the NH_4_^+^:NO_3_^−^ Ratio on the Photosynthetic Capacity

**Figure 3:**
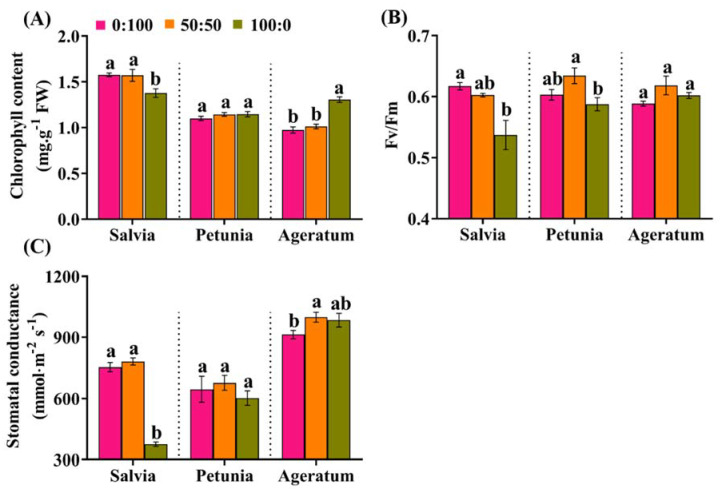
(**A**) Chlorophyll contents (**B**) Fv/Fm and (**C**) stomatal conductance of salvia, petunia, and ageratum plants as affected by three different NH_4_^+^:NO_3_^−^ ratios; data are the mean of six independent replicates ±SD; significant differences at *p* less than 0.05 are denoted by different letters according to the one-way ANOVA (Duncan’s multiple range test).

The NH_4_^+^ responses by salvia, petunia, and ageratum have been preliminary determined by the growth characteristics described above. To better understand and confirm the tolerances of these three bedding plants to NH_4_^+^, we further assessed the photosynthetic capacity in response to the increasing NH_4_^+^ concentrations. The photosynthetic capacity was characterized and determined herein in terms of three parameters: chlorophyll contents, Fv/Fm values, and stomatal conductance. As is apparent in Figure 3A, salvia plants solely fed with NH_4_^+^ had a lower chlorophyll content than those cultured with other NH_4_^+^:NO_3_^−^ ratios, while a considerably higher chlorophyll content was monitored under identical circumstances in ageratum.

Similarly, the Fv/Fm ratio, an indicator of the maximal photochemical efficiency, showed significant variations according to the species and the NH_4_^+^:NO_3_^−^ ratio. In comparison to salvia plants grown with 0:100 or 50:50 NH_4_^+^:NO_3_^−^, the Fv/Fm ratio in those grown with 100:0 NH_4_^+^:NO_3_^−^ was dramatically reduced to 0.53 merely (Figure 3B ‘Salvia part’); petunia plants supplied with 0:100 NH_4_^+^:NO_3_^−^ and 100:0 NH_4_^+^:NO_3_^−^ displayed varying degrees of decrease compared to that grown with 50:50 NH_4_^+^:NO_3_^−^ (Figure 3B ‘Petunia part’); ageratum plants fed solely with NH_4_^+^ had a slightly higher Fv/Fm than those solely fed with NO_3_^−^, although ageratum grown with 50:50 NH_4_^+^:NO_3_^−^ had the greatest Fv/Fm value (Figure 3B ‘Ageratum part’).

Furthermore, the stomatal conductance of the three tested species displayed an identical pattern to that of the chlorophyll content and Fv/Fm ratio. The stomatal conductance of salvia treated with 0:100 and 50:50 NH_4_^+^:NO_3_^−^ had 2.08-fold and 2.01-fold enhancement compared with those grown with 100:0 NH_4_^+^:NO_3_^−^ (Figure 3C ‘Salvia part’); no significant differences in the stomatal conductance were detected in petunia plants in response to the different NH_4_^+^:NO_3_^−^ ratios (Figure 3C ‘Petunia part’); however, we found that ageratum plants grown with the 100:0 NH_4_^+^:NO_3_^−^ experienced a 7.76% increase in the stomatal conductance relative to the ageratum plants grown with 0:100 NH_4_^+^:NO_3_^−^ (Figure 3C ‘Ageratum part’).

#### 2.2.2. Effects of the NH_4_^+^:NO_3_^−^ Ratio on the Contents of Nitrogen and Carbohydrates

The uptake of nitrogen (N) and sufficient supply of carbohydrates (C) are warranted for NH_4_^+^ assimilation. We therefore measured, compared, and analyzed the contents of N and C in the three species in response to the increasing NH_4_^+^ concentration.

**Figure 4:**
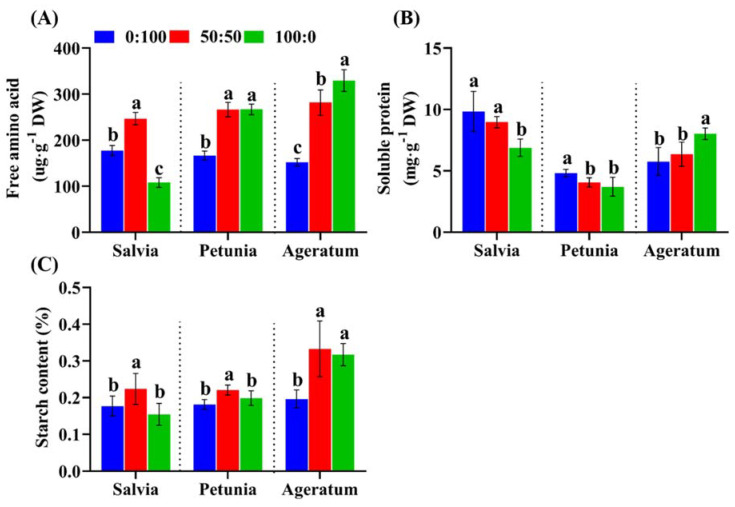
(**A**) Free amino acid contents (**B**) soluble protein contents and (**C**) starch contents in salvia, petunia, and ageratum as affected by three different NH_4_^+^:NO_3_^−^ ratios; data are the means of six independent replicates ±SD; significant differences at *p* less than 0.05 are denoted by different letters according to the one-way ANOVA (Duncan’s multiple range test).

When the external NH_4_^+^ concentration increased from 0% to 50%, the free amino acid contents showed a parallel increase, regardless of the species; however, the free amino acid contents in petunia and ageratum kept growing whereas those in salvia notably dropped when the NH_4_^+^ concentration increased from 50% to 100% (Figure 4A). On the contrary, a progressive decline of the soluble protein content was observed in salvia and petunia as the NH_4_^+^ concentration increased, but a gradual increase in the soluble protein content was observed in ageratum in response to an increasing NH_4_^+^ concentration (Figure 4B).

The C status was estimated herein by the starch contents assay. Clearly, great differences in the starch content was observed in response to the three NH_4_^+^:NO_3_^−^ ratios, irrespective of the species (Figure 4C). In specific, plants grown under increasing NH_4_^+^ concentrations from 0% to 50% possessed a parallel increase in the starch content, which was determined to be 26.00%, 22.10%, and 69.90% by salvia, petunia, and ageratum, respectively; in contrast, as the NH_4_^+^ concentration increased from 50% to 100%, the starch content decreased by 30.94%, 9.95%, and 4.80% in salvia, petunia, and ageratum, respectively.

Accordingly, the results of *2.1* and *2.2* showed that salvia and petunia were extremely sensitive and moderately sensitive, respectively, to high NH_4_^+^ concentrations, whereas ageratum was highly tolerant to NH_4_^+^, but very sensitive to high NO_3_^−^ concentrations.

### 2.3. Activities of Major Enzymes Involved in the NH_4_^+^ Assimilation Pathway in Leaves

After NH_4_^+^ is taken up by the roots, certain key enzymes regarding GS, GOGST, and GDH implicated in the NH_4_^+^ incorporations and assimilations. To figure out the NH_4_^+^ tolerance mechanisms, we compared the actions and changes of these major enzymes in the three species studied.

**Figure 5:**
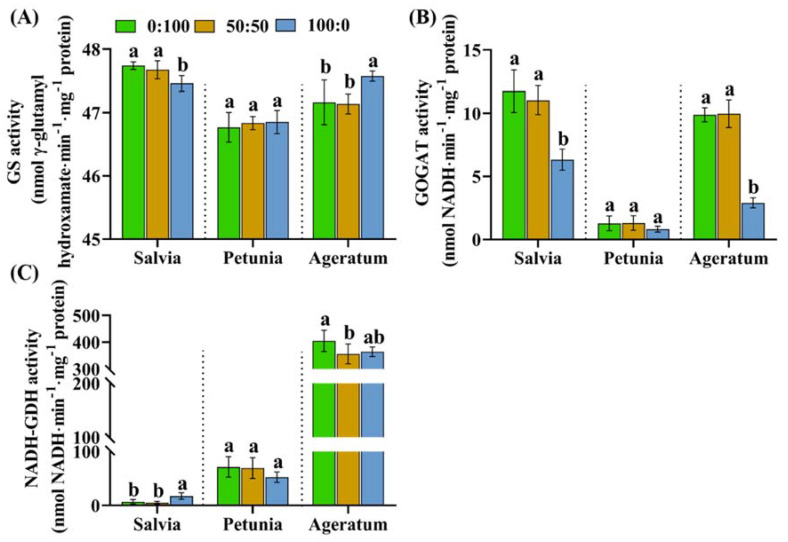
(**A**) Leaf GS activities, (**B**) leaf GOGAT activities, and (**C**) leaf NADH-dependent GDH activities in salvia, petunia, and ageratum as affected by three NH_4_^+^:NO_3_^−^ ratios; data are the means of six independent replicates ±SD; significant differences at *p* less than 0.05 are denoted by different letters according to the one-way ANOVA (Duncan’s multiple range test).

GS, GOGAT, and NADH-dependent GDH activities were individually measured in the leaves of salvia, petunia, and ageratum after they were cultivated with three different NH_4_^+^:NO_3_^−^ ratios. As shown in Figure 5, the activities of these three enzymes behaved differently among the species and NH_4_^+^:NO_3_^−^ ratio, especially the comparisons undertaken between salvia and ageratum plants exclusively supplied with NH_4_^+^ or NO_3_^−^; petunia plants did not experience significant differences in these enzyme activities regardless of the NH_4_^+^:NO_3_^−^ ratio.

As presented in Figure 5A, irrespective of the species and NH_4_^+^:NO_3_^−^ ratio, the GS activity showed minor differences (only ranging from 46.77 to 47.74 nmol γ-glutamyl hydroxamate per minute^−1^ mg^−1^ protein); a slight decrease was found in salvia as the NH_4_^+^ concentration increased from 0% to 100%, while negligible increases were monitored in petunia and ageratum.

Unexpectedly, the GOGAT activities in plants progressively declined as the level of NH_4_^+^ increased, regardless of the species (Figure 5B); still, taking the petunia aside, the NADH-dependent GDH activities in salvia and ageratum sharply and slightly boosted, respectively, as the NH_4_^+^ concentration increased from 50% to 100%. Interestingly, ageratum displayed the greatest GDH activities among all the treatments, followed in order by petunia and salvia (Figure 5C).

### 2.4. Activities of Major Enzymes Involved in the NH_4_^+^ Assimilation Pathway in Roots

Similar to the quantifications of GS, GOGAT, and NADH-dependent GDH activities in leaves, we further tested and analyzed their activities in the roots for a better understanding on the NH_4_^+^ detoxification mechanism based on the differences in the NH_4_^+^ tolerance.

**Figure 6:**
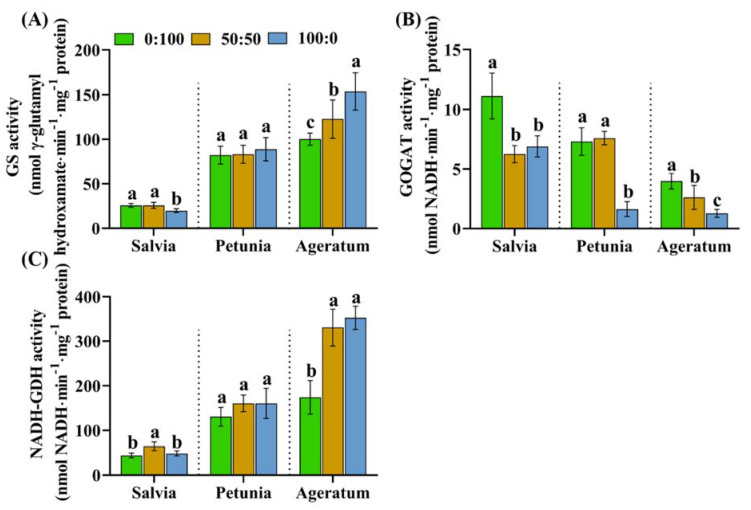
(**A**) Root GS activities, (**B**) root GOGAT activities, and (**C**) root NADH-dependent GDH activities in salvia, petunia, and ageratum as affected by three NH_4_^+^:NO_3_^−^ ratios; data are the means of six independent replicates ±SD; significant differences at *p* less than 0.05 are denoted by different letters according to the one-way ANOVA (Duncan’s multiple range test).

The GS, GOGAT, and NADH-GDH activities in the roots of the three bedding plants were significantly influenced by the NH_4_^+^:NO_3_^−^ ratio. On average, the activities of GS and NADH-GDH were observed to be positively correlated with the NH_4_^+^ tolerance, whereas the GOGAT activities were negatively regulated by the NH_4_^+^ tolerance (Figure 6). In addition, the summed GS activities in ageratum roots appeared to be two-fold higher than that in leaves; while declined activities were detected in salvia roots compared to that in leaves (Figure 5A and Figure 6A). The NADH-GDH activity in ageratum roots showed a NH_4_^+^ dose-enhancing tendency, whereas a NH_4_^+^ dose-reducing tendency was monitored in ageratum leaves (Figure 5C and Figure 6C).

50:50 NH_4_^+^:NO_3_^−^ yielded the most active GS and NADH-GDH in salvia roots, whereas petunia and ageratum plants cultured with 100:0 NH_4_^+^:NO_3_^−^ displayed the highest such activities in the roots (Figure 6A,C). Specifically, for salvia, in comparison with the plants grown with 50:50 NH_4_^+^:NO_3_^−^, the GS and NADH-GDH activities in roots treated with 100:0 NH_4_^+^:NO_3_^−^ decreased by 23.09% and 24.63%, respectively (Figure 6A,C ‘Salvia part’). However, more importantly, the GS and NADH-GDH activities in solely NH_4_^+^-fed ageratum roots increased by 25.24% and 6.64%, respectively, as compared to those in response to 50:50 NH_4_^+^:NO_3_^−^ (Figure 6A,C ‘Ageratum part’). In general, the GOGAT activity was notably dropped as the NH_4_^+^ concentration increased from 50% to 100% (Figure 6B).

## 3. Discussion

It has been widely accepted that ammonium is strongly phytotoxic above a certain threshold concentration. However, this threshold on manifested NH_4_^+^ toxicity vary greatly among species [22]. Few researchers have elucidated the ammonium tolerance mechanisms in bedding plants. Thus, we hypothesized that comparisons and analysis between the NH_4_^+^-sensitive species and NH_4_^+^-tolerant species may dissect the distinct strategies that are responsible for the ammonium tolerance.

### 3.1. Preliminary NH_4_^+^ Tolerance Determination by Plant Growth Investigations

In this study, we chose to test the NH_4_^+^ tolerances in three bedding plants: salvia, petunia, and ageratum, in terms of the responses toward an increasing NH_4_^+^ concentration. Consequently, the shoot and root growth were greatly affected by not only the three NH_4_^+^:NO_3_^−^ ratios, but also the species. An addition of NH_4_^+^ to plants cultured with NO_3_^−^ showed the best growth, compared to exclusive supply of NH_4_^+^ or NO_3_^−^, as displayed by ameliorated plant growth and root system (Figure 1 and Figure 2; Table 1). More importantly, salvia plants grown with 100:0 NH_4_^+^:NO_3_^−^ were remarkably stunted, as characterized by the development of NH_4_^+^ toxicity symptoms accompanied by leaf tip burn, chlorosis, and necrosis (Figure 1D). In contrast, sole NH_4_^+^ supply suppressed the petunia growth to a relative minor extent, while solely NH_4_^+^-fed ageratum possessed the greatest whole plant weight and leaf traits (leaf length, width, and area) (Figure 1E; Table 1). Interestingly, ageratum treated with 50:50 NH_4_^+^:NO_3_^−^ had a better root system (root volume and root surface area) than when grown with 100:0 NH_4_^+^:NO_3_^−^, which can be explained by the fact that exogenous NH_4_^+^ is sensed by the leaves only after saturating the roots [8,23]. Accordingly, the preliminary data suggested that salvia was the most sensitive to high NH_4_^+^ concentrations, followed by petunia, but ageratum was tolerant to high NH_4_^+^ levels.

### 3.2. NH_4_^+^ Tolerance Confirmnations with the Photosynthestic Capacity and N-C Distributions

To further confirm the NH_4_^+^ tolerances in salvia, petunia, and ageratum, how the three NH_4_^+^:NO_3_^−^ regimes influenced photosynthesis and distributions of nitrogen (N) and carbohydrates (C) was studied.

The photosynthetic capacity was presented herein by the contents of chlorophyll contents, Fv/Fm ratio, and stomatal conductance. Plenty of previous studies have suggested that a higher level of chlorophyll content delivered a greater light absorption rate and further promoted an increase in photosynthesis [24,25]. The present study showed that solely NH_4_^+^-fed ageratum plants had the highest contents of chlorophyll, while solely NH_4_^+^-fed salvia plants had sharply declined chlorophyll contents (Figure 3A). The Fv/Fm reflected the state of photosystem II (PSII), where a low ratio at less than 0.6 is widely adopted for the early stress indication [26]. Indeed, solely NH_4_^+^-fed salvia and petunia as well as solely NO_3_^−^-fed ageratum had Fv/Fm values that indicated stresses (Figure 3B), which also indicated that salvia and petunia were NH_4_^+^-sensitive and ageratum was NO_3_^−^-sensitive. Meanwhile, pioneer researchers demonstrated that a reduction of the stomatal conductance primarily resulted in the decrease of the CO_2_ transport efficiency, thus leading to the decline of the photosynthetic rate [27,28,29]. Clearly, the greatest stomatal conductance was conferred by 50:50 NH_4_^+^:NO_3_^−^, irrespective of the species. However, the stomatal conductance was the most significantly inhibited when salvia was exposed to 100:0 NH_4_^+^:NO_3_^−^, followed by petunia; no major differences in the stomatal conductance were observed in ageratum (Figure 3C). Succinctly, ageratum exhibited a greater photosynthetic capacity than petunia, followed by salvia, when supplied 100:0 NH_4_^+^:NO_3_^−^.

Ammonium toxicity developed by plants was not only linked to the photosynthetic efficiency, but also to the depletion of the carbon skeleton [8,9,30]. Sufficient internal C supply for excessive NH_4_^+^ assimilation and a better maintenance of the C-N equilibrium were vital strategies for ammonium detoxification [9,31,32]. Exposed to high external NH_4_^+^ concentrations, ammonium-tolerant species were observed to exhibit a strong ability for incorporating NH_4_^+^ into amino acids [15,16,33]. In our trials, it is worthy to note that the free amino acid contents were positively correlated with the NH_4_^+^ concentration in ageratum. However, salvia plants grown with 100:0 NH_4_^+^:NO_3_^−^ failed to adequately convert NH_4_^+^ into amino acids (Figure 4A). Besides, we noticed that the soluble protein contents were negatively correlated with the A-N ratio in salvia and petunia, but not in ageratum (Figure 4B), which was attributed to the high NO_3_^−^ sensitivity of ageratum. In addition, sole NH_4_^+^ supply markedly decreased the starch contents merely in salvia (Figure 4C), because of poor capacity on the removal of excess NH_4_^+^, leading to a higher cost of carbohydrate use [8,34].

### 3.3. NH_4_^+^ Tolerances of Salvia, Petunia, Ageratum

Therefore, we identified and characterized that salvia and petunia were, respectively, highly sensitive and moderately sensitive, while ageratum was tolerant, to high ammonium concentrations. Oppositely, ageratum was determined to show sensitivity to high nitrate concentrations. These findings were in agreement with those in numerous previous works [35,36,37].

### 3.4. Explorations of the NH_4_^+^ Tolerance Mechanisms in the Three Bedding Plants

Ammonium assimilation was mainly carried out via two pathways: the major one is GS/GOGAT, and the other alternative route is GDH [12,13,14,15,38,39]. Despite the considerable progress that have been made in explaining the ammonium assimilation mechanisms, controversy still exists on the priorities of the ammonium assimilation routes, and the corresponding plant parts, for different plant species.

#### 3.4.1. Leaf GS, GOGAT, and NADH-GDH Activities Are Negligible in the Contributing NH_4_^+^ Tolerances of the Three Bedding Plants

Our data displayed that the extremely NH_4_^+^-sensitive salvia failed to enhance, but progressively diminished the leaf GS activity as the NH_4_^+^ concentration increased from 50% to 100%, whereas the moderately NH_4_^+^-sensitive petunia and NH_4_^+^-tolerant ageratum both boosted the leaf GS activities in response to the increasing NH_4_^+^ supply, which suggested that the leaf GS activity acted as an important factor for the NH_4_^+^ assimilation. This phenomenon has also been frequently reported in other species [15,40,41,42]. However, the value of the leaf GS activity was altered by only one unit of nmol γ-glutamyl hydroxamate per minute^−1^ mg^−1^ protein, which was negligible compared with the root GS activity (Figure 5A and Figure 6A). In addition, the concerted action of the GS and GOGAT pathways played key roles in the glutamate synthesis when the NH_4_^+^ was limited [43]. This was confirmed again in this study where GOGAT showed the opposite regulatory patterns with GS (Figure 5B and Figure 6B), indicating that GOGAT was not referred in the NH_4_^+^ tolerance of bedding plants. Unexpectedly, the leaf NADH-GDH activities in ageratum was lower in solely NH_4_^+^-fed plants than that in solely NO_3_^−^-fed plants, but curiously, solely NH_4_^+^-fed salvia plants increased the leaf NADH-GDH activities to cope with the increasing NH_4_^+^ concentrations (Figure 5C), probably because a slight remainder of NH_4_^+^ was translocated to ageratum leaves [21,44]. Accordingly, the leaf GS, GOGAT, and NADH-GDH were negligibly relevant to contribute to the NH_4_^+^ tolerance of the three bedding plants.

#### 3.4.2. Root GS, NADH-GDH Activities Play Important Roles in Enhancing the NH_4_^+^ Tolerance of the Three Bedding Plants

In contrast to the GS and NADH-GDH activities in the leaves, considerable changes in roots were recorded regardless of the species and NH_4_^+^:NO_3_^−^ ratio in the present study (Figure 6A,C). We noticed varying degrees of increases in the root GS and NADH-GDH activities when the NH_4_^+^ concentration increased from 0% to 50%. Elevated activities of GS and NADH-GDH were required in the assimilations of the excessive NH_4_^+^ to prevent toxicity, consistent with the results regarding certain studies in vegetables [41,45], crops [15,46], and *Arabidopsis* [11,47]. As the NH_4_^+^ concentration increased from 50% to 100%, the greatest reinforcements of both the root GS and NADH-GDH activities were monitored in the NH_4_^+^-tolerant species ageratum, followed by the moderately NH_4_^+^-sensitive species petunia, but in the extremely NH_4_^+^-sensitive salvia, root GS and NADH-GDH activities were not significantly affected and in some cases even reduced, in response to high NH_4_^+^ concentrations (Figure 6A,C). Furthermore, salvia grown with 100:0 NH_4_^+^:NO_3_^−^ developed ammonium toxicity symptoms, probably due to the low activities of root GS and NADH-GDH (Figure 1D and Figure 6A,C ‘Salvia part’). These results led to the hypothesis that, in the roots of petunia and ageratum, the GS and NADH-GDH acted as a detoxification strategy under extremely high NH_4_^+^ concentrations [15,16,48]. Ageratum stimulated greater GS and GDH activities in the roots in response to increasing NH_4_^+^, thereby conferring an enhanced NH_4_^+^ tolerance; whereas in petunia and salvia, neither the root GS nor NADH-GDH activities were significantly increased and in some cases even decreased in the roots, resulting in poor NH_4_^+^ tolerances. Accordingly, the root GS and NADH-GDH are corroborated to be, at least in the three bedding plants tested, closely associated with the NH_4_^+^ tolerance enhancement.

## 4. Materials and Methods

### 4.1. Plant Materials and Culture Conditions

The experiments were initially performed during the growing season and repeated in the following fall during 2021 at Gyeongsang National University (35°88′ N, 128°01′ E, Jinju, Gyeongsang nam-do, Korea), under a fiberglass greenhouse condition with a 13-h photoperiod. The average day and night temperatures were 23.6 °C/20.3 °C. Seeds of the three bedding plants (*Ageratum houstonianum* ‘Aloha Blue’, *Petunia hybrida* ‘Madness Red’, and *Salvia splendens* ‘Vista Red’) were purchased from Pan American Seeds Company (West Chicago, IL 60185, USA), planted into 200-cell plug trays filled with a commercial growing medium (Bas Van Buuren Substrate, EN-12580, De Lier, The Netherlands), and germinated under a mist propagation tunnel. The seedlings were transferred to a metal bench and were allowed to grow for 10 days.

### 4.2. Ammonium-Nitrate Ratio Treatments

Subsequently, similarly sized seedlings with two fully expanded true leaves were monitored and subjected to solutions with the different NH_4_^+^:NO_3_^−^ ratios. Three NH_4_^+^:NO_3_^−^ ratios, namely, 0:100, 50:50, and 100:0 were formed at a constant nitrogen supply of 13.0 meq·liter^−1^ on the basis of a multipurpose nutrient solution (MNS), which has been reported by our pioneer research [45]. For each species, a completely randomized design was employed with three biological replicates per treatment, consisting of 60 plants.

### 4.3. Ammonium Tolerance Determinations

To determine the ammonium tolerance of the three bedding plants, certain vital growth attributes, including shoot-related traits (leaf length and width, leaf area, and chlorosis appearance) and root morphology parameters (root length, volume, and root surface area) of juvenile plants in response to increasing NH_4_^+^ concentration in the nutrient solutions, were investigated via destructive sampling. Specifically, the leaf area data were collected with a leaf area apparatus (LI-3000, Lincoln, NE, USA). The root morphological parameters were analyzed with the WinRhizo Pro image system (2007a, Regent Instruments, Sainte-Foy, QC, Canada) linked to a professional scanner (Expression 1000XL, Epson America Inc., Long Beach, CA, USA).

### 4.4. Further Confirmations of the Ammonium Tolerance

The ammonium tolerance of the three bedding plants were further characterized and reconfirmed herein by the examinations of the photosynthetic capacity (chlorophyll, Fv/Fm, stomatal conductance), nitrogen contents (Free amino acid and total protein contents), and carbohydrates contents (starch content). Briefly, contents of chlorophyll a and b were spectrophotometrically monitored and calculated according to a protocol presented by Arnon [49]; the Fv/Fm and stomatal conductance assays were carried out on the mid-lamina portion of fully expanded leaves from 9:00 to 11:30 with a FluorPen FP 100 (Photon Systems Instruments, Drásov, Czech Republic) and a Decagon Leaf Porometer (SC-1, Decagon Device, Pullman, USA), respectively. During harvest, the plants were sampled by separating the shoots and roots, individually labeled, carefully collected, quickly frozen in liquid N_2_, and placed at −80 °C for further experiments.

The free amino acid content was quantified based on the nihydrin method [50]. An accurate 0.3 g fine frozen powder was mixed in 3 mL of 10% acetic acid and the solution was adjusted to 20 mL by adding distilled water, after which 1 mL filtrate was obtained through filtration and mixed thoroughly with 5 mL of a nihydrin reaction buffer containing 2% nihydrin and 0.25% ascorbic acid (prepared daily); the total protein content was estimated by using the Bradford reagent [51]. Ca. 100 mg samples were homogenized in a 1.5 mL characterized PBS (50 mM, 1 mM EDTA, 1 mM polyvinylpyrolidone and 0.05% triton-X, pH = 7.0) over ice, the protein crude extracts were acquired after centrifugation (12,000 rpm, 4 °C, 20 min) and used for the protein content assay.

An anthrone sulfuric acid colorimetry [52] with minor modifications was adopted for the determination of the starch content. In brief, a total of 0.3 g finely ground samples were mixed vigorously with 25 mL of deionized water and then subjected to a water bath at 95 °C for at least 50 min, and the residue was collected after centrifugation at 6500 rpm for 10 min and reaction with 2 mL of 9.6 M perchloric acid (HClO_4_). The mixture was subsequently incubated in boiling water for 30 min. Total of 5 mL of concentrated sulfuric acid (H_2_SO_4_) was slowly added to the starch solution after homogenization with 0.5 mL of 2% anthrone. The absorbance of the mixed solution was finally read by spectrophotometric absorbance at 485 nm.

### 4.5. Determination of the Key Enzymatic Activities in the N Metabolism Pathways

Activities of the key enzymes for N assimilation GS (glutamine synthetase), GOGAT (glutamate synthetase) and NADH-dependent GDH (glutamate dehydrogenase) were determined in the leaves and roots.

Total of 0.5 g of frozen plant sample was finely ground in a pre-cooled mortar and homogenized in a 3 mL extraction solution (0.05 mol/L Tris-HCl, 2 mmol/L MgSO_4_, 2 mmol/L DDT, 0.4 mol/L sucrose). The supernatant was obtained after centrifugation (13,000 rpm, 4 °C, 20 min) and used for the subsequent GS, GOGAT, and GDH activity assays.

The GS activity was estimated in the leaves and roots following an approach introduced by Oaks et al. [53]. In brief, a mixture of 0.7 mL of crude enzyme extract and 2.3 mL of the reaction medium (containing 0.1 M Tris-HCl, 20 mM sodium glutamate and cysteine, 2 mM EGTA, 80 mM Mg^2+^ and hydroxylamine hydrochloride, and 40 mM daily made ATP) was incubated in a water bath at 37 °C for 25 min. After incubation, the reaction was stopped immediately by adding 1 mL of 0.37 M FeCl_3_ in 0.6 M HCl. After shaking for 5 min and centrifugation for 10 min (5000 g, Rt), the absorbance of the supernatant was spectrophotometrically read at 540 nm. The GS activity was characterized as the synthesis of one nmol γ-glutamyl hydroxamate min^−1^ mg^−1^ protein.

The GOGAT activity was assessed in the leaves and roots based on the method of Lin [54]. The reaction was started by adding 0.5 mL crude enzyme extract to a mixture containing 0.1 mL of 10 mM KCl, 0.05 mL of 0.1 M α-oxoglutarate, 0.4 mL of 20 mM L-glutamine, 0.2 mL of 3 mM NADH, and 3 mL of 25 mM Tris-HCl (pH 7.6). The change of absorbance was spectrophotometrically recorded at 340 nm. One unit of GOGAT activity was defined as a decrease at 0.001 of absorbance per minute.

The NADH-dependent GDH activity was assayed in the leaves and roots in accordance to a method reported by Kanamori et al. [55]. The assay mixture was made up of a 2.5 mL buffer (15.4 mM Tris-HCl, 23.1 mM of α-Ketoglutarate, 231 mM of NH_4_Cl, pH 8.0), 0.3 mL distilled water, 0.1 mL 30 mM CaCl_2_, and 6 mM NADH. The reaction was triggered by adding 0.1 mL crude enzyme extract, and the decrease in the absorbance was spectrophotometrically monitored at 340 nm after a 3-min water bath at 30 °C. One unit of GDH activity was defined as the oxidation of nmol reduced NADH protein mg^−1^ min^−1^.

### 4.6. Statistical Analysis and Graphing

All the measurements were taken with size-independent replicates. The SAS statistical software (Version 8.2 Inst., Cary, NC, USA) was adopted for statistical analysis. Differences among the mean values was regarded as significant when the probability (*p*) from one-way ANOVA (analysis of variance) was ≤0.05, followed by Duncan’s multiple range test. Graphs were drawn via the GraphPad Prism 8.0 program.

## 5. Conclusions

To sum up, among the three bedding plants tested, this study identifies that salvia and petunia are highly and moderately NH_4_^+^-sensitive species, respectively, while ageratum is a NH_4_^+^-tolerant species. As primarily evidenced by the growth responses under increasing NH_4_^+^ concentrations, further assessments on the photosynthetic capacity and nitrogen (N)-carbohydrate (C) distributions not only confirmed the NH_4_^+^ tolerances of the three bedding plants, but also showed the influences regarding different forms of N supply on the N metabolism. More importantly, the quantifications and comparisons of the major enzymes in the ammonium assimilation pathways in the three bedding plants suggested that the GS and NADH-GDH in roots, not in leaves, probably underpin the ammonium tolerances of bedding plants, delivering a promising insight for examining the ammonium tolerance of other bedding plant species.

## Figures and Tables

**Table 1:** Growth parameters of salvia, petunia, and ageratum treated with three different NH_4_^+^:NO_3_^−^ ratios.

Species (A)	Treatment (B)	Shoot Length (cm)	Leaf Length (cm)	Leaf Width (cm)	Leaf Area (cm^2^)	Total Root Length (cm)
Salvia	0:100	3.58a ^y^	2.37a	2.03a	3.57a	126.32a
	50:50	3.47a	2.38a	2.08a	3.63a	147.05a
	100:0	2.15b	1.98b	1.63b	2.81b	89.99b
Petunia	0:100	0.72a	2.43ab	1.35a	2.30b	46.42a
	50:50	0.75a	2.60a	1.43a	3.05a	50.50a
	100:0	0.65a	2.18b	1.17b	2.11b	43.59a
Ageratum	0:100	3.95b	1.82b	1.63b	4.07b	94.74b
	50:50	5.05a	2.48a	2.25a	5.02b	170.04a
	100:0	4.57a	2.50a	2.37a	6.13a	109.61b
*F*-test	A	*** ^z^	*	***	***	***
	B	***	***	***	***	***
	A × B	***	***	***	***	***

^y^ Data are the means ± SD (n = 6 independent replicates) accompanied by different lowercase letters that indicate significant differences at *p* ≤ 0.05; ^z^ the *F*-test values generated from the ANOVA of species and treatments refer to * *p* ˂ 0.05 and *** *p* ˂ 0.001.

## References

[1] Kaur G., Asthir B., Bains N., Farooq M. (2015). Nitrogen nutrition, its assimilation and remobilization in diverse wheat genotypes. Int. J. Agric. Biol..

[2] Dobermann A.R. (2005). Nitrogen use efficiency-state of the art. Agron. Fac. Publ..

[3] Choudhury A., Kennedy I. (2005). Nitrogen fertilizer losses from rice soils and control of environmental pollution problems. Commun. Soil Sci. Plan..

[4] Jackson L.E., Burger M., Cavagnaro T.R. (2008). Roots, nitrogen transformations, and ecosystem services. Annu. Rev. Plant Biol..

[5] Maynard D., Barker A., Minotti P., Peck N. (1976). Nitrate accumulation in vegetables. Adv. Agron..

[6] Li B., Li G., Kronzucker H.J., Baluška F., Shi W. (2014). Ammonium stress in *Arabidopsis*: Signaling, genetic loci, and physiological targets. Trends Plant Sci..

[7] Esteban R., Ariz I., Cruz C., Moran J.F. (2016). Mechanisms of ammonium toxicity and the quest for tolerance. Plant Sci..

[8] Britto D.T., Kronzucker H.J. (2002). NH_4_^+^ toxicity in higher plants: A critical review. J. Plant Physiol..

[9] Wang H., Ni L., Xie P. (2013). The mitigating effect of calcification-dependent of utilization of inorganic carbon of Chara vulgaris Linn on NH_4_-N toxicity. Chemosphere.

[10] Britto D.T., Siddiqi M.Y., Glass A.D., Kronzucker H.J. (2001). Futile transmembrane NH_4_^+^ cycling: A cellular hypothesis to explain ammonium toxicity in plants. Proc. Natl. Acad. Sci. USA.

[11] Hachiya T., Inaba J., Wakazaki M., Sato M., Toyooka K., Miyagi A., Kawai-Yamada M., Sugiura D., Nakagawa T., Kiba T. (2021). Excessive ammonium assimilation by plastidic glutamine synthetase causes ammonium toxicity in *Arabidopsis thaliana*. Nat. Commun..

[12] Castro-Rodríguez V., García-Gutiérrez A., Canales J., Avila C., Kirby E.G., Cánovas F.M. (2011). The glutamine synthetase gene family in *Populus*. BMC Plant Boil..

[13] McAllister C.H., Beatty P.H., Good A.G. (2012). Engineering nitrogen use efficient crop plants: The current status. Plant Biotechnol. J..

[14] Lightfoot D., Baron A., Wootton J. (1988). Expression of the Escherichia coli glutamate dehydrogenase gene in the cyanobacterium Synechococcus PCC6301 causes ammonium tolerance. Plant Mol. Boil..

[15] Cruz C., Bio A., Domínguez-Valdivia M., Aparicio-Tejo P.M., Lamsfus C., Martins-Louçao M.A. (2006). How does glutamine synthetase activity determine plant tolerance to ammonium?. Planta.

[16] Xian L., Zhang Y., Cao Y., Wan T., Gong Y., Dai C., Ochieng W.A., Nasimiyu A.T., Li W., Liu F. (2020). Glutamate dehydrogenase plays an important role in ammonium detoxification by submerged macrophytes. Sci. Total Environ..

[17] El Omari R., Rueda-López M., Avila C., Crespillo R., Nhiri M., Cánovas F.M. (2010). Ammonium tolerance and the regulation of two cytosolic glutamine synthetases in the roots of sorghum. Funct. Plant Biol..

[18] Nelson P.V., Song C.-Y., Huang J., Niedziela C.E., Swallow W.H. (2012). Relative effects of fertilizer nitrogen form and phosphate level on control of bedding plant seedling growth. HortScience.

[19] Gibson J.L., Nelson P.V., Pitchay D.S., Whipker B.E. (2001). Identifying nutrient deficiencies of bedding plants. NC. State Univ. Floric. Res. Florex.

[20] Jeong B., Lee C.W. (1992). Ammonium and nitrate nutrition of 11 bedding plant species. Acta Hort..

[21] Xu G., Fan X., Miller A.J. (2012). Plant nitrogen assimilation and use efficiency. Annu. Rev. Plant Biol..

[22] Domínguez-Valdivia M.D., Aparicio-Tejo P.M., Lamsfus C., Cruz C., Martins-Loução M.A., Moran J.F. (2008). Nitrogen nutrition and antioxidant metabolism in ammonium-tolerant and-sensitive plants. Physiol. Plant..

[23] Luo J., Li H., Liu T., Polle A., Peng C., Luo Z.-B. (2013). Nitrogen metabolism of two contrasting poplar species during acclimation to limiting nitrogen availability. J. Exp. Bot..

[24] Bowes G., Ogren W., Hageman R. (1972). Light Saturation, photosynthesis rate, RuDP carboxylase activity, and specific leaf weight in soybeans grown under different light intensities 1. Crop Sci..

[25] Buttery B., Buzzell R. (1977). The relationship between chlorophyll content and rate of photosynthesis in soybeans. Can. J. Plant Sci..

[26] Saeck E.A., Brien K.R.O., Burford M.A. (2016). Nitrogen response of natural phytoplankton communities: A new indicator based on photosynthetic efficiency Fv/Fm. Mar. Ecol. Prog. Ser..

[27] Shang H., Shen G. (2018). Effect of ammonium/nitrate ratio on pak choi (*Brassica chinensis* L.) photosynthetic capacity and biomass accumulation under low light intensity and water deficit. Photosynthetica.

[28] Torralbo F., González-Moro M.B., Baroja-Fernández E., Aranjuelo I., González-Murua C. (2019). Differential regulation of stomatal conductance as a strategy to cope with ammonium fertilizer under ambient versus elevated CO_2_. Front. Plant Sci..

[29] Ibrahim M.H., Jaafar H.Z.E., Karimi E., Ghasemzadeh A. (2012). Primary, Secondary Metabolites, Photosynthetic Capacity and Antioxidant Activity of the Malaysian Herb Kacip Fatimah (*Labisia Pumila* Benth) Exposed to Potassium Fertilization under Greenhouse Conditions. Int. J. Mol. Sci..

[30] Bittsánszky A., Pilinszky K., Gyulai G., Komives T. (2015). Overcoming ammonium toxicity. Plant Sci..

[31] Schortemeyer M., Stamp P., Feil B. (1997). Ammonium tolerance and carbohydrate status in maize cultivars. Ann. Bot..

[32] Cao T., Xie P., Ni L., Zhang M., Xu J. (2009). Carbon and nitrogen metabolism of an eutrophication tolerative macrophyte, Potamogeton crispus, under NH_4_^+^ stress and low light availability. Environ. Exp. Bot..

[33] Jiranek V., Langridge P., Henschke P. (1995). Amino acid and ammonium utilization by Saccharomyces cerevisiae wine yeasts from a chemically defined medium. Am. J. Enol. Viticult..

[34] Kirkby E. (1968). Influence of Ammonium and Nitrate Nutrition on the Cation-Anion Blance and Nitrogen and Carbohydrate Metabolism of White Mustard Plants Grown in Dilute Nutrient Solutions. Soil Sci..

[35] Jeong B.R., Lee C.W. (1992). Growth suppression and raised tissue Cl-contents in NH_4_^+^-fed Marigold, Petunia, and Salvia. J. Am. Soc. Hortic. Sci..

[36] Nelson P.V., Niedziela C.E., Pitchay D.S., Mingis N.C. (2010). Effectiveness, ammonium impact and potassium adequacy of soybean-base liquid fertilizer on bedding plants. J. Plant Nutr..

[37] Jeong B.R., Lee C.W. (1996). Influence of ammonium, nitrate, and chloride on solution pH and ion uptake by ageratum and salvia in hydroponic culture. J. Plant Nutr..

[38] Ireland R.J., Lea P.J. (1998). The Enzymes of Glutamine. Glutamate, Asparagine, and Aspartate Metabolism. Plant Amino Acids.

[39] DeLuna A., Avendaño A., Riego L., González A. (2001). NADP-glutamate dehydrogenase isoenzymes of Saccharomyces cerevisiae: Purification, kinetic properties, and physiological roles. J. Biol. Chem..

[40] Lasa B., Frechilla S., Lamsfus C., Aparicio-Tejo P. (2001). The sensitivity to ammonium nutrition is related to nitrogen accumulation. Sci. Hortic..

[41] Horchani F., Hajri R., Aschi-Smiti S. (2010). Effect of ammonium or nitrate nutrition on photosynthesis, growth, and nitrogen assimilation in tomato plants. J. Plant Nutr. Soil Sci..

[42] Wei Y., Wang X., Zhang Z., Xiong S., Meng X., Zhang J., Wang L., Zhang X., Yu M., Ma X. (2020). Nitrogen regulating the expression and localization of four glutamine synthetase isoforms in wheat (*Triticum aestivum* L.). Int. J. Mol. Sci..

[43] Guillamón J.M., van Riel N.A., Giuseppin M.L., Verrips C.T. (2001). The glutamate synthase (GOGAT) of Saccharomyces cerevisiae plays an important role in central nitrogen metabolism. FEMS Yeast Res..

[44] Black B.L., Fuchigami L.H., Coleman G.D. (2002). Partitioning of nitrate assimilation among leaves, stems and roots of poplar. Tree Physiol..

[45] Song J., Yang J., Jeong B.R. (2021). Growth, Quality, and Nitrogen Assimilation in Response to High Ammonium or Nitrate Supply in Cabbage (*Brassica campestris* L.) and Lettuce (*Lactuca sativa* L.). Agronomy.

[46] Wang F., Gao J., Tian Z., Liu Y., Abid M., Jiang D., Cao W., Dai T. (2016). Adaptation to rhizosphere acidification is a necessary prerequisite for wheat (*Triticum aestivum* L.) seedling resistance to ammonium stress. Plant Physiol. Bioch..

[47] Sarasketa A., González-Moro M.B., González-Murua C., Marino D. (2014). Exploring ammonium tolerance in a large panel of *Arabidopsis thaliana* natural accessions. J. Exp. Bot..

[48] Lea P.J., Miflin B.J. (2003). Glutamate synthase and the synthesis of glutamate in plants. Plant Physiol. Bioch..

[49] Arnon D.I. (1949). Copper enzymes in isolated chloroplasts. Polyphenoloxidase in *Beta vulgaris*. Plant Physiol..

[50] Yokoyama S., Hiramatsu J.-I. (2003). A modified ninhydrin reagent using ascorbic acid instead of potassium cyanide. J. Biosci Bioeng..

[51] Hammond J.B., Kruger N.J. (1988). The bradford method for protein quantitation. New Protein Techniques.

[52] McCready R., Guggolz J., Silviera V., Owens H. (1950). Determination of starch and amylose in vegetables. Anal. Chem..

[53] Oaks A., Stulen I., Jones K., Winspear M.J., Misra S., Boesel I.L. (1980). Enzymes of nitrogen assimilation in maize roots. Planta.

[54] Lin C.C., Kao C.H. (1996). Disturbed ammonium assimilation is associated with growth inhibition of roots in rice seedlings caused by NaCl. Plant Growth Regul..

[55] Kanamori T., Konishi S., Takahashi E. (1972). Inducible formation of glutamate dehydrogenase in rice plant roots by the addition of ammonia to the media. Physiol. Plant..

